# Potential of Essential Oils and Major EO Constituents in the Chemical Control of *Spodoptera frugiperda*

**DOI:** 10.3390/plants14142204

**Published:** 2025-07-16

**Authors:** Virginia Lara Usseglio, Magalí Beato, José Sebastián Dambolena, María Paula Zunino

**Affiliations:** 1 Instituto Multidisciplinario de Biología Vegetal (IMBiV-CONICET-UNC), Córdoba X5000, Argentina; mbeato@imbiv.unc.edu.ar (M.B.); jdambolena@imbiv.unc.edu.ar (J.S.D.); paula.zunino.254@unc.edu.ar (M.P.Z.); 2 Cátedra de Química Orgánica y Productos Naturales (FCEFyN-UNC), Córdoba X5000, Argentina; 3 Instituto de Ciencia y Tecnología de los Alimentos (ICTA-FCEFyN-UNC), Córdoba X5000, Argentina

**Keywords:** *Spodoptera frugiperda*, natural products, biopesticides

## Abstract

*Spodoptera frugiperda* is a major agricultural pest worldwide, causing significant economic loss to maize crops. Its control largely depends on synthetic pesticides, which contribute to resistance development, harm non-target organisms, and lead to environmental degradation. Essential oils and their main components offer a more sustainable and ecologically sound alternative for the management of *S. frugiperda*. This study evaluated the effects of selected essential oils and their bioactive compounds on the survival and behavior of *S. frugiperda* using toxicity and preference assays. Peppermint essential oil and its major constituent, pulegone, significantly reduced the survival of *S. frugiperda*, with effects similar to those caused by synthetic insecticides. Eucalyptus essential oil and its main compound, 1,8-cineole, also influenced the behavior of *S. frugiperda*, suggesting potential for use in repellents. These findings highlight the use of essential oils and their main constituents/active constituents as bioinsecticides and their integration into environmentally friendly pest management strategies.

## 1. Introduction

Maize (*Zea mays* L.) is one of the most important crops worldwide and a crucial component in animal feed and industrial applications [1]. In the 2023/24 season, world corn production was 1224.3 million metric tons, the main cereal harvested, followed by wheat and rice [2,3]. Its production is threatened by various biotic and abiotic stresses, among which insect pests play a major role in yield reduction and quality deterioration [4]. The fall armyworm (*Spodoptera frugiperda*), a highly destructive lepidopteran pest, has gained global notoriety due to its rapid spread and devastating effects on maize crops [5,6].

*Spodoptera frugiperda* is a polyphagous insect that primarily targets maize but can also infest a wide range of crops, including rice, sorghum, and cotton [7]. Its ability to migrate long distances, coupled with its high reproductive rate and adaptability to environmental conditions, has allowed its spread [8,9,10]. The larval stages of *S. frugiperda* feed on maize leaves, stems, and reproductive structures, leading to severe defoliation and reduced photosynthetic efficiency, which results in significant yield loss [4,7].

Synthetic chemical insecticides have been widely used as a primary control strategy [4,11]. These pesticides have provided effective pest management and have helped sustain maize production in many regions. However, its misuse has raised serious concerns over its effects on human health, environmental sustainability, non-target organism toxicity, and the development of resistance in *S. frugiperda* populations [11]. Resistance to insecticides such as pyrethroids, organophosphates, and neonicotinoids has been reported in multiple regions, reducing the efficacy of conventional control methods and increasing the reliance on higher doses or more potent chemicals.

Exploring environmentally friendly and sustainable options for crop protection has thus become increasingly urgent. One promising alternative is the use of plant-derived bioactive compounds, particularly essential oils (EOs). EOs are complex mixtures of volatile secondary metabolites extracted from aromatic plants and are known for their insecticidal, repellent, and antifeedant properties [12,13,14]. The application of EOs in pest management has gained increasing attention due to their biodegradability, low toxicity to non-target organisms, and potential to reduce dependency on synthetic pesticides [15]. These natural compounds represent promising and sustainable tools for reducing *S. frugiperda* populations while minimizing environmental and health risks associated with conventional pesticides [16,17,18,19,20,21]. Therefore, this study aims to evaluate the effects of EOs and their major components on the survival and attraction/repellence response of *S. frugiperda*.

## 2. Results and Discussion

### 2.1. Essential Oil Composition

Table 1 presents the major compounds identified by GC-MS in Peppermint, Eucalyptus, and Orange EOs. In the case of Peppermint EO (*Minthostachys verticillata*), the main constituent identified was pulegone (~69%) as the predominant compound, followed by menthone (~21%). For orange EO (*Citrus sinensis*), limonene was the principal component, accounting for approximately 97% of the composition, while the remaining 3% consisted of compounds present at concentrations below 1%. Finally, the main compound found in eucalyptus EO (*Eucalyptus globulus*) was 1,8-cineole (~81%), followed by α-pinene and o-cymene at lower percentages (≅7%).

### 2.2. Insecticidal Activity

**Figure 1:**
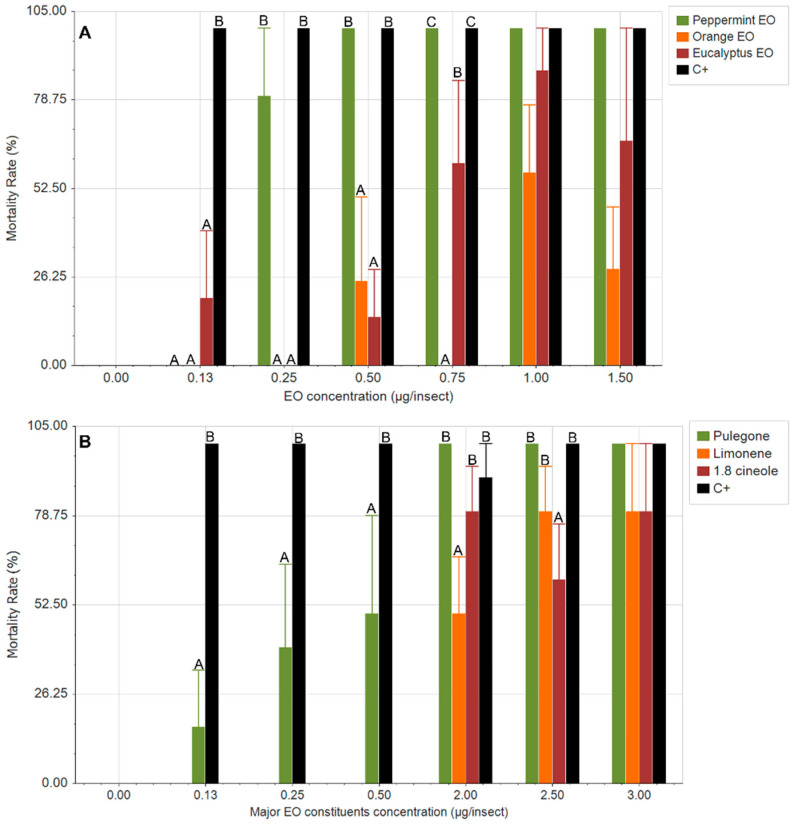
Toxic effects of essential oils (**A**) and their major compounds (**B**) against the *S. frugiperda* second larval stage. C+: Positive control for toxicity (commercial insecticide Zamuray10^®^). Different letters indicate statistical differences between treatments, as determined by ANOVA and DGC tests (*p* < 0.05).

Figure 1 shows the contact effect of EOs and their major constituents on the survival of second-instar *S. frugiperda* larvae. The most effective EO was Peppermint, reaching 100% mortality at 0.5 μg/insect, followed by Eucalyptus, which did not exceed 80% mortality at the highest concentration tested (Figure 1A). On the other hand, the effect of the major compounds studied showed that pulegone had the greatest toxicity (Figure 1B), lower than that exhibited by the EO, indicating that the mixture of compounds has a synergistic effect on the survival of *S. frugiperda*. Authors have found this harmful effect of pulegone not only in toxicity tests but also in antifeedant assays [24,25,26]. The highly toxic effects of pulegone, and therefore of Peppermint EO, may be due to alpha and beta unsaturation and the carbonyl group in this molecule. In a previous study, we found that chemical compounds with these characteristics exerted significant harmful effects not only on *Spodoptera* species but also on other insect pests [10,27,28].

As 1,8-cineole and limonene, the predominant compounds in Eucalyptus and Orange Eos, respectively, exhibited similar effects, which were notably lower than those in pulegone (Figure 1B). In the case of 1,8-cineole, the mortality rate at the tested concentrations was similar to that observed for the highest concentration of Eucalyptus EO, which could be attributed to the action of this pure compound. In Orange EO, mortality did not exceed 50%, which was similar to that of pure limonene. As a positive control for toxicity, the synthetic insecticide Zamuray 10^®^ was used, whose active ingredient is bifenthrin (10% *w*/*v*). As shown in Figure 1, mortality caused by this insecticide was 100% at all concentrations tested. In the case of Peppermint EO, a comparable effect was observed at 0.5 μg/insect, as previously noted, whereas for pulegone, it was not achieved until 2 μg/insect.

The present study demonstrates the insecticidal potential of EOs and their major compounds against second-instar *S. frugiperda* larvae. Among the tested EOs, Peppermint EO showed the highest larvicidal activity, achieving complete mortality at concentrations as low as 0.5 μg/insect. This finding highlights the potent bioactivity of Peppermint EO, which appears comparable to that of the synthetic insecticide bifenthrin, used as a positive control. In addition, Eucalyptus EO also demonstrated notable activity, although it did not exceed 80% mortality, even at the highest concentration tested. This difference in efficacy between Peppermint and Eucalyptus EOs may be attributed to differences in their chemical composition, particularly the relative concentration and type of active organic compounds present.

Although pulegone was the most toxic pure compound evaluated, its efficacy remained lower than that of the complete Peppermint EO, suggesting a synergistic effect of its components that enhances its insecticidal activity. This synergism agrees with that in previous reports, indicating that complex mixtures in EOs can act more effectively than isolated components due to additive or synergistic interactions [29]. For example, menthone, the second most abundant compound in this EO, has demonstrated toxic effects on various insects [30,31,32], suggesting that its presence may enhance the insecticidal activity of pulegone. In contrast, 1,8-cineole and limonene displayed significantly lower toxicity. The mortality caused by 1,8-cineole was comparable to that observed at the highest concentration of Eucalyptus EO, suggesting that the activity of Eucalyptus EO may be largely attributed to this compound. Compounds such as *α*-pinene and *o*-cymene show low or negligible toxicity against *S. frugiperda* larvae, confirming that the observed effect is primarily attributable to the presence of 1,8-cineole [10]. However, this was not the case for Orange EO, as both essential oil and pure limonene caused less than 50% mortality, indicating limited insecticidal efficacy. Although the effects of orange EO and limonene have limited effects on insect survival, studies have shown that both significantly reduce nutrient reserves (lipids, proteins, sugars, and glycogen), decreasing fertility, longevity, and anti-feeding effects [33,34]. Hence, different studies should be conducted to evaluate their potential for controlling this pest.

Overall, these results underscore the potential of Peppermint EO as a natural, plant-based insecticidal agent against *S. frugiperda*, offering an effective and environmentally friendly alternative to conventional synthetic insecticides. Future studies should focus on characterizing the interaction between the components in EOs to better understand and harness their synergistic properties for pest control.

### 2.3. Attraction/Repellence Response

**Figure 2:**
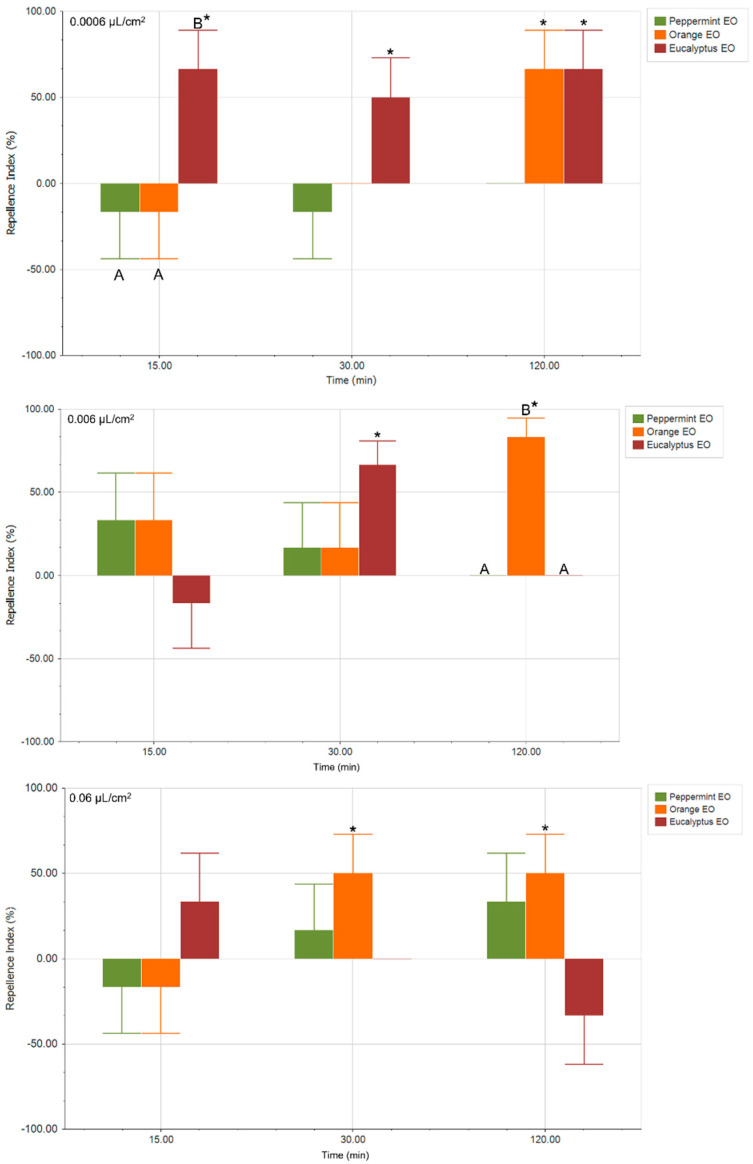
Repellence Index (%) of *S. frugiperda*, second larval stage, exposed to essential oils three times (15, 30, and 120 min). Positive values indicate repellence, and negative values indicate attraction. * refers to statistical differences for a paired *t*-test (*p* < 0.05), and different letters denote statistical differences between RI by ANOVA and DGC test (*p* < 0.05).

To evaluate the effect of EOs and their main components on the preference of *S. frugiperda*, attraction/repellency bioassays were conducted in a circular arena. Figure 2 shows the repellence index (RI) obtained after exposing this insect to EOs at three concentrations (0.0006, 0.006, and 0.06 µL/cm^2^) and at three points (15, 30, and 120 min of exposure). At the lowest concentration (0.0006 µL/cm^2^), Eucalyptus EO was the only treatment that produced a statistically significant repellent effect on insect behavior at the three times evaluated (*p*-value < 0.05), with a repellence index (RI) of 66.67 ± 25.62%, a trend that remained consistent after 30 min exposure (RI: 50.00 ± 26.91%, *p*-value < 0.05) and 120 min exposure (RI: 66.67 ± 25.69%, *p*-value < 0.05). After 120 min exposure, Orange EOs also exhibited statistically significant repellent effects, reaching the same level of repellency as that of Eucalyptus EO. As concentration increased, variations in insect response were observed. Larvae of *S. frugiperda* exposed to 0.006 µL/cm^2^ exhibited no defined behavioral response at shorter exposure times to the selected EOs. However, after 30 min, Eucalyptus EO showed a repellent effect (RI: 66.67 ± 23.57%, *p*-value < 0.05), which disappeared after 120 min of exposure. At 120 min, only Orange EO induced a significant behavioral effect, showing a repellent response (RI: 83.33 ± 25.46%, *p*-value < 0.05), as also observed at the lowest concentration (0.0006 µL/cm^2^). Finally, at the highest concentration (0.06 µL/cm^2^), only Orange EO produced a statistically significant response from *S. frugiperda*, being repellent after 30 and 120 min exposure (RI: 50.00 ± 26.69% and RI: 50.00 ± 26.75%, respectively).

**Figure 3:**
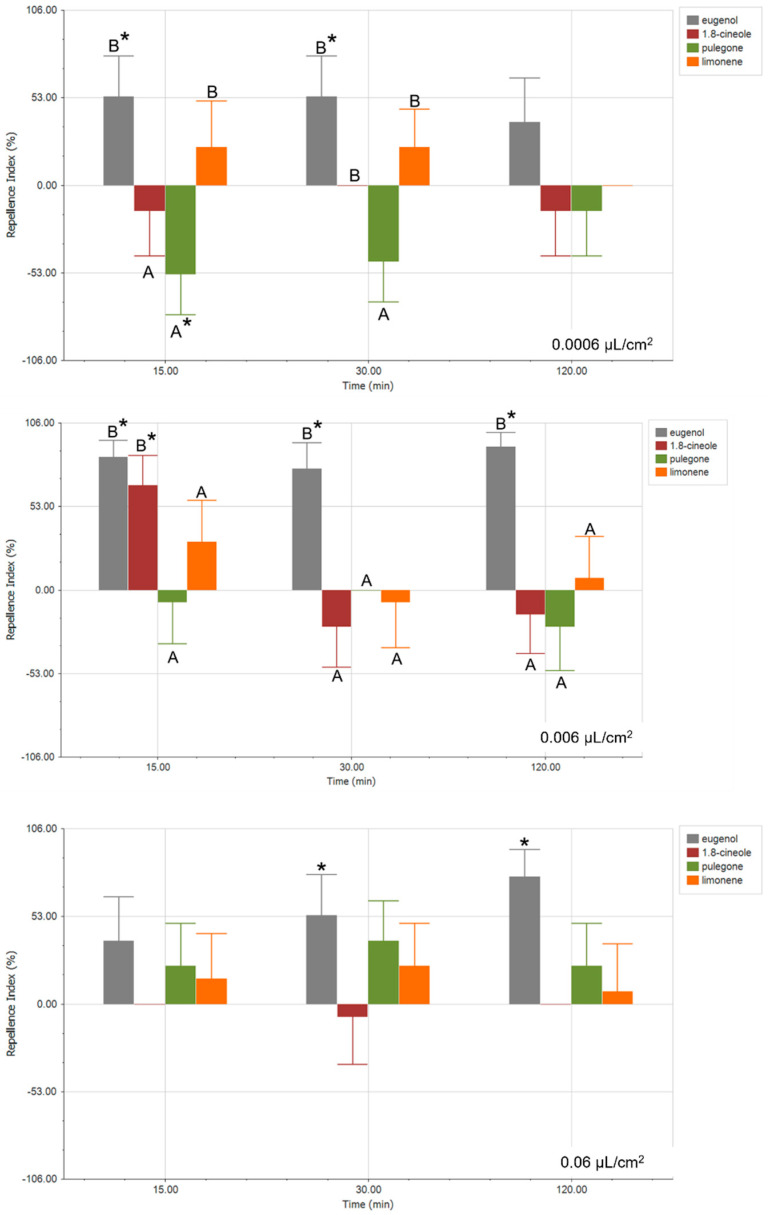
Repellence Index (%) of *S. frugiperda*, second larval stage, exposed to major EO constituents three times (15, 30, and 120 min). Positive values indicate repellence, and negative values indicate attraction. * refers to statistical differences for a paired *t*-test (*p* < 0.05), and different letters denote statistical differences between RI by ANOVA and DGC test (*p* < 0.05).

In parallel, the effect of the major EO compounds on the behavior of *S. frugiperda* was evaluated using the same concentrations and exposure times as described previously (Figure 3). Eugenol was used as a positive control for repellence [35,36,37]. At the lowest tested concentration (0.0006 µL/cm^2^), pulegone showed an attractive trend, which was statistically significant only after 15 min exposure (RI: −53.85 ± 26.09%, *p*-value < 0.05). In contrast to the results observed for 1,8-cineole, a statistical behavioral response was noted from *S. frugiperda* at this concentration. However, at 0.006 µL/cm^2^, 1,8-cineole showed a repellent effect after 15 min of exposure (RI: 66.67 ± 22.30%, *p*-value < 0.05), while the attractant effect previously observed for pulegone at 0.0006 µL/cm^2^ was not replicated. At all concentrations tested, limonene showed no statistically significant effects on the behavior of *S. frugiperda*; however, a repellent tendency of this compound was observed. Additionally, at 0.06 µL/cm^2^, none of the tested compounds showed a clear behavioral response. Notably, under this condition, the repellent effect of eugenol, used as a positive control, was also absent at 15 min, suggesting a potential methodological issue related to the rapid saturation of organic compound vapors in the headspace of the device used, which could hinder the selection of insects.

The behavioral assays conducted in this study provide valuable insights into the olfactory-mediated responses of *S. frugiperda* larvae to selected EOs and their major constituents. Overall, our results demonstrate that both the composition and concentration of EOs and their constituents play a central role in determining their attractant or repellent effects on lepidopteran pests.

At low concentrations (0.0006 µL/cm^2^), Eucalyptus EO exhibited a statistically significant repellent effect after both 15 and 30 min exposure, a trend that was not observed at higher concentrations or longer exposure times. This repellent response of Eucalyptus EO was also reported by Wang et al. (2023) [20] in larvae and adults of *S. frugiperda*, as well as by other authors in larvae and adults of other lepidopteran species [38,39,40], indicating that the repellent effect of this EO may be preserved across this group of insect pests. These authors also noted a repellent effect of Lemon EO [20], whose main component, as in Orange EO, is limonene, suggesting that the effect observed in our study may be attributed to high percentages of this compound in its composition. The concentration-dependent response of EO may reflect a natural ecological interaction, where lower concentrations of major EO compounds mimic host plant volatiles, while higher concentrations may confuse or repel insects due to olfactory saturation or toxic effects [41]. Interestingly, 1,8-cineole did not replicate the repellent effect of the whole oil at the same concentration, suggesting a synergistic interaction among the constituents of EOs [24,42]. This underlines the importance of evaluating both whole EOs and isolated components, as the biological activity of EOs is often not attributable to a single compound [29].

The repellent effect of Orange EO, particularly at longer exposure times and higher concentrations (e.g., 0.06 µL/cm^2^ at 120 min), suggests that specific terpenes in this oil may have a prolonged stimulating effect on larval olfaction. This consistent repellence at low and high concentrations underscores its potential as a repellent pest management strategy. In contrast, pulegone showed a significant attractant effect at low concentrations and short exposure times; yet this response was not maintained at higher doses or with prolonged exposure, indicating that its attractant properties may be transient or subject to rapid desensitization in the larval olfactory system. Wang et al. (2023) [20] reported a repellent effect of Peppermint EO that we have been unable to determine in our work; however, lack of information on the scientific name of the species used and its main components introduces uncertainty about whether the extract originated from the same plant species, limiting comparison of results.

The behavioral neutrality observed at the highest concentration tested (0.06 µL/cm^2^) for all compounds suggests that olfactory saturation or vapor-phase interference may have affected larval choice. Rapid saturation of the arena’s headspace may have caused a uniform distribution of volatiles, preventing larvae from detecting a concentration gradient and thus impairing their ability to choose between odor sources. This finding underlines the need to optimize experimental conditions, particularly in behavioral assays involving volatile compounds, where air circulation and spatial distribution are crucial.

Our findings emphasize the complexity of insect–plant chemical interactions and the context-dependent nature of behavioral responses to semiochemicals. Although certain EOs and their major components are promising behavioral modulators of *S. frugiperda*, future research should further explore the mechanisms of olfactory reception, potential habituation effects, and the interaction between EO constituents. This knowledge is essential for the development of effective and sustainable pest management strategies based on natural products.

## 3. Materials and Methods

### 3.1. EOs and Major EO Constituents

The EOs used in this work were Orange (EO) (*Citrus sinensis* L.), Peppermint (EO) (*Minthostachys verticillata* Griseb.), and Eucalyptus EO (*Eucalyptus globulus* Labill.). Peppermint and Eucalyptus EOs were provided by a group of local producers from Valle de Calamuchita, Córdoba, Argentina, and were obtained from aerial parts by hydrodistillation using a Clevenger apparatus. Orange EO was obtained by cold pressing the peels and supplied by ECA Agroindustria (Concordia, Entre Ríos, Argentina). To determine the composition of each EO, chromatographic analysis was performed using a Perkin-Elmer Clarus 600 gas chromatograph coupled with an ion trap mass detector (GC-MS). EOs were diluted in hexane (1:10 *v*/*v*), and 1 μL of solution was injected. An ELITE 5 ms capillary column (60 m × 0.25 mm i.d. and 0.25 µm coating thickness) was employed for separating the individual components. The chromatographic conditions were as follows: Injector at 250 °C in splitless mode; oven temperature programming: 50 °C (2 min)–5 °C/min (1 min)–240 °C (5 min); total run time 45 min; and constant detector temperature of 240 °C. Helium served as the carrier gas (flow rate = 1.5 mL/min), and compounds were identified by comparing their retention indices and mass spectra with published data and available libraries (NIST, Gaithersburg, MD, USA [43]).

**Figure 4:**
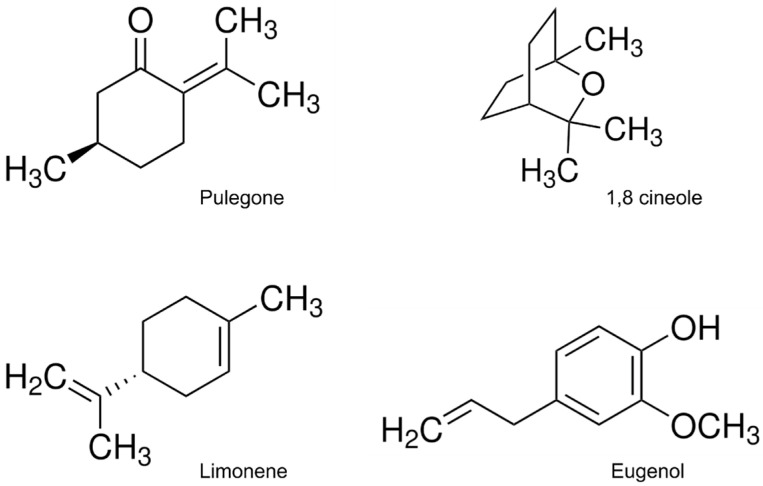
Molecular structure of major EO constituents.

Most EO constituents used in this investigation were (Figure 4): ^®^-2-Isopropylidene-5-methylcyclohexanone ((+)-pulegone), 1,3,3-Trimethyl-2-oxabicyclo[2.2.2]octane (1,8-cineole), (R)-4-Isopropenyl-1-methyl-1-cyclohexen^®^(R)-(+)-limonene), and 2-Methoxy-4-(2-propenyl)phenol (eugenol). All organic compounds were purchased from Merck^®^ (Buenos Aires, Argentina). Zamuray 10 (2-methylbiphenyl-3-ylmethyl (Z)-(1RS)-cis-3-(2-chloro-3,3,3-trifluoroprop-1-enyl)-2,2-dimethylcyclopropanecarboxylate, bifenthrin) from Gleba^®^, a commercial insecticide, was used as a positive control.

### 3.2. Insects

*Spodoptera frugiperda* larvae were maintained under controlled laboratory conditions (28 ± 2 °C and 70 ± 5% relative humidity (RH)). Second-instar larvae were used according to the optimal stage for chemical control of *S. frugiperda*, as determined by Insect Resistance Management (IRM) and the Program and Insecticide Resistance Action Committee [10,44].

The larvae were fed an artificial diet containing the following main ingredients: bean flour (150 g), wheat germ (35 g), yeast (40 g), vitamins (2 g), agar (40 g), methylparaben (3 g), sodium benzoate (3 g), sorbic acid (1 g), ascorbic acid (6 g), streptomycin (0.16 g), oxytetracycline (2.16 g), formaldehyde 40% *v*/*v* (2 mL), and distilled water (1 L).

### 3.3. Topical Application Bioassay

The toxicity of EOs and their major constituents was determined according to Silva Bibiano et al. (2022) [45]. Briefly, EOs and organic compounds were solubilized in acetone to generate stock solutions. Then, 1 µL of the solution was topically administered with a Hamilton syringe into the second thoracic segment of the larvae. The concentrations tested were 0.13, 0.25, 0.75, 1.0, and 1.5 μg/insect for EOs and 0.13, 0.25, 0.5, 2.0, 2.5, and 3.0 μg/insect for pure compounds. For the negative control, 1 μL of acetone was topically applied to each larva. The positive control consisted of the commercial insecticide Zamuray 10, applied at the same concentrations as those used for EOs and pure compounds. The larvae were then separated and placed with food in Petri dishes to prevent cannibalism, and mortality was recorded at 24 h [45]. The experiments were performed at 28 ± 2 °C and 70 ± 5% RH. Five replicates for each treatment were performed twice.

### 3.4. Attractant/Repellent Activity Assay

The effect of EOs and their major constituents on *Spodoptera frugiperda* preference was determined following Beato et al. (2022) [46] with some modifications. Briefly, a Whatman filter paper disk (6 cm diameter) was placed at the bottom of a glass Petri dish and separated into two halves. Increasing concentrations of EOs or organic compounds were added to half of the paper disk (0.06, 0.006, and 0.0006 μL/cm^2^) and the other half was treated with acetone (control). Each half of the paper disk was embedded in 150 μL of solution (acetone for control and EO or organic compound diluted in acetone for treatments) and left for 5 min until the solvent evaporated. For each petri dish, one *S. frugiperda* larva deprived of food was released, and its position (control or treatment) was recorded at 15, 30, and 120 min. The experiment was performed at 28 ± 2 °C and 70 ± 5% RH. A repellence index (RI) was calculated according to Nerio et al. (2009) [47] with some modifications:

$$RI=\left(C-T\right)\times 100$$

where T represents the number of insects on the treated half of the plate, and C represents the number of insects in the control half. Negative RI values indicate attraction, and positive values indicate repellence [47]. All experiments were carried out five times and twice per concentration, and the position of the treatments was varied. The position of each larva (control or treatment) was determined individually.

### 3.5. Statistical Analysis

The statistical difference in the mortality rate was determined using ANOVA. A paired *t*-test was used to determine the statistical difference in the choice of insects, and ANOVA was used for comparison of RIs. The assumptions of normality and homogeneity of variance were tested. These analyses were carried out with the statistical software Navure^®^ (Córdoba, Argentina).

## 4. Conclusions

This study highlights the dual bioactivity of selected EOs and their major constituents in *Spodoptera frugiperda* larvae, demonstrating both lethal and behavioral effects. Peppermint EO has emerged as the most effective insecticidal agent, achieving 100% larval mortality at low doses, an effect largely attributed to pulegone and the synergistic interactions among its constituents.

Behavioral assays revealed that Eucalyptus and Orange EOs acted as effective repellents, particularly at low concentrations and longer exposure times. Notably, pulegone exhibited a transient attractive effect, stressing the importance of exposure time and concentration in modulating larval behavior.

Overall, these findings underscore the potential of Peppermint and Eucalyptus EOs as natural alternatives for managing *S. frugiperda* through toxicity and behavioral disruption. Further research should explore formulation strategies, field validation, and the role of synergistic effects in developing eco-friendly pest management tools. The results of this study could contribute to the formulation of eco-friendly pest control strategies that align with sustainable agricultural principles, ultimately reducing the ecological footprint of maize protection practices.

## Figures and Tables

**Table 1:** Relative percentage concentrations of the constituents of Eucalyptus, Peppermint, and Orange essential oils (EOs) according to their elution order in gas chromatography analysis.

Calculated Retention Index	Theoretical Retention Index [22] *	Compounds	Eucalyptus EO	Peppermint EO	Orange EO	Methods of Identification
924	930	*α*-thujene	nd	tr	tr	GCMS
930	939	*α*-pinene	7.33	0.22	0.44	GCMS-Co
969	975	sabinene	1.82	0.11	0.22	GCMS
972	979	*β*-pinene	1.32	0.29	tr	GCMS-Co
987	991	*β*-myrcene	Tr	0.21	1.35	GCMS-Co
1008	999	octanal	nd	nd	tr	GCMS
1020	1026	*o*-cymene	7.73	nd	tr	GCMS
1024	1029	limonene		0.11	**97.26**	GCMS-Co
1028	1031	1,8-cineole	**81.80**	nd	nd	GCMS-Co
1032	1037	*cis-β*-ocimene	nd	1.7	nd	GCMS
1044	1050	*trans*-*β*-ocimene	nd	0.62	nd	GCMS
1054	1060	*γ*-terpinene	nd	tr	nd	GCMS
1095	1097	linalool	nd	0.38	0.22	GCMS-Co
1107	1101	nonanal	nd	nd	tr	GCMS
1146	1142	*trans*-limonene oxide	nd	nd	tr	GCMS
1148	1153	menthone	nd	21.42	nd	GCMS-Co
1158	1163	isomenthone	nd	0.49	nd	GCMS
1165	1188 [23] **	*trans*-isopulegone	nd	0.60	nd	GCMS
1186	1189	*α*-terpineol	nd	0.13	tr	GCMS-Co
1205	1202	decanal	nd	nd	0.16	GCMS
1233	1237	pulegone	nd	**69.2**	nd	GCMS-Co
1239	1243	carvone	nd	nd	tr	GCMS-Co
1249	1253	piperitone	nd	0.49	nd	GCMS
1417	1419	*β*-caryophyllene	nd	0.25	tr	GCMS-Co
1433	1434	gurjunene	nd	0.33	nd	GCMS
1484	1485	germacrene D	nd	2.34	tr	GCMS
**Total**			**100**	**98.89**	**100**	

tr: Stands for trace amounts of the compound and is used when the average amount of a particular compound is <0.05%. nd: Not determined. GCMS: Peak identification based on MS comparison with file spectra. Co: Peak identification based on a standard comparison with relative retention time. * Theoretical RI from Adams (2007) [22]. ** RI calculated by López et al. (2022) [23].

## Data Availability

The raw data supporting the conclusions of this article will be made available by the authors on request (vusseglio@imbiv.unc.edu.ar).
